# Facile In Situ Preparation and In Vitro Antibacterial Activity of PDMAEMA-Based Silver-Bearing Copolymer Micelles

**DOI:** 10.1186/s11671-019-3074-z

**Published:** 2019-07-27

**Authors:** Wenjing Lin, Kaihang Huang, Yanzhe Li, Yanlin Qin, Di Xiong, Jiabao Ling, Guobin Yi, Zilun Tang, Jinglian Lin, Yunwei Huang, Chufen Yang, Jufang Wang

**Affiliations:** 1 School of Chemical Engineering and Light Industry, Guangdong University of Technology, Guangzhou, 510006 People’s Republic of China; 2 School of Bioscience & Bioengineering, South China University of Technology, Guangzhou, 510640 People’s Republic of China; 3 School of Chemical Engineering, Xiangtan University, Xiangtan, 411105 China

**Keywords:** AgNPs, Polymer micelles, Star polymer, PDMAEMA, Antimicrobials

## Abstract

Well-defined polymer micelles with core-shell structure are good delivery platform for stabilizing silver nanoparticles (AgNPs) in the field of antimicrobials targeting diseases. The rational construction of the polymer structure, an efficient, facile, and green preparation approach, and comprehensive exploration of the derived AgNPs are necessary, such as size, particle stability, antibacterial activity, and other properties. Herein, we designed and assessed the in vitro antimicrobial activity of AgNPs-decorated copolymer micelles with different copolymer topologies. First, linear or four-arm star triblock copolymers with the similar molecular weight and degree of polymerization were obtained, which consisted of DMAEMA for in situ reduction of silver ions to form AgNPs without external reducing agent. HEMA and PEGMA in micellar shell gave an enhanced stability of AgNPs during blood circulation. The combination of computational modeling and experimental results indicated that both types of micelles could fabricate AgNPs with monodisperse and spherical morphology. Star copolymer micelles stabilized AgNPs had smaller average size, better stability, and higher antibacterial activity than those with linear structure, which may due to higher stability of micelles from star copolymers. Furthermore, the cytotoxicity evaluation test showed that the achieved linear or star copolymers micelles stabilized AgNPs had good biocompatibility. This work provides a facile and universal approach in the rational design of micelles stabilized AgNPs with suitable topology for fighting against a wide range of bacterial infections.

**Electronic supplementary material:**

The online version of this article (10.1186/s11671-019-3074-z) contains supplementary material, which is available to authorized users.

## Introduction

In the past few decades, a slate of traditional antimicrobial agents has been extensively used to treat infectious diseases. According to the World Health Organization, rapid emergence of multidrug-resistant microorganisms has become an increasingly serious global problem, which has ranked in top three in the list of major threats to human health [1–5]. Therefore, it is necessary to develop new antimicrobial agents with good safety, effective antibacterial ability without producing bacterial resistance. Silver nanoparticles (AgNPs) as one of the best antimicrobial agents since ancient times, were widely used in consumer goods because of their superior performance against various bacterial and fungal pathogens, relatively low toxicity to mammalian cells, and limited bacterial resistance [6–10]. AgNPs are able to improve membrane permeability of bacteria, permeate into cytoplasm, denaturalize bacterial proteins, and disrupt the replication of bacterial, resulting in the death of bacterial [11–13]. A large number of silver formulations have been employed to elucidate the antibacterial activity of AgNPs [14–17], for example, a wound dressing through a zwitterionic polycarboxybetaine hydrogel along with antibacterial AgNPs as core component proposed by Zhang et al*.* [18], the multifunctional surfaces obtained via multicomponent coating for co-immobilizing AgNPs proposed by Moreno-Couranjou et al., etc. [19]*.*

However, large specific surface area and high surface energy led to the aggregation of AgNPs, which has become a big bottleneck for their application. Thus, polymer matrix or external stabilizer is needed in order to stabilize the AgNPs. As known, polymer matrix is the most common method to solve the aggregation problem. At present, several methods were used to stabilize AgNPs with polymer matrix, such as chemical reduction method, electrochemical method, photochemical method, and microwave method. Among them, chemical reduction is a common and effective method. Silver nitrate is reduced into AgNPs by adding reductants such as hydrazine hydrate (N_2_H_4_), sodium borohydride (NaBH_4_), sodium citrate, and ascorbic acid in solution [20–23]. For example, Hoda et al. fabricated polystyrene-block-polyacrylicacid (PS-*b*-PAA) reverse micelles loaded with the 20 nm AgNPs under the influence of the reducing agent N_2_H_4_, and the PS blocks played the outer layer in toluene [24]. Liu’s group reported that self-assembled micelles nanotemplates were prepared from poly(ε-caprolactone)-block-poly (aspartic acid) (PCL-*b*-PAsp). Well-dispersed AgNPs were prepared with AgNO_3_ as the precursor and NaBH_4_ as the reducer [25]. However, the above methods were not environment friendly, and the addition of excessive reductants produce by-products, making it difficult to purify AgNPs and restricting their application of antimicrobials targeting infectious diseases.

In the meanwhile, it has been reported that polymers containing amine group could be used as both reductant and stabilizer to prepare AgNPs in situ. For example, Lang et al. synthesized six-arm star polymers consisting of PCL, 2-(dimethylamino) ethyl methacrylate (DMAEMA), and poly (ethylene glycol) methyl ether methacrylate (PEGMA). The system directly reduced silver nitrate into AgNPs without adding any other reductant in aqueous phase [26]. Although the AgNPs mentioned above present facile surface modification without additional reducer, relative to the gold nanoparticles [27, 28], the effect of polymer topologies on the reduction and stability of silver nanoparticles along with their application in the micelles-based antibacterial activity is less studied.

**Scheme 1:**
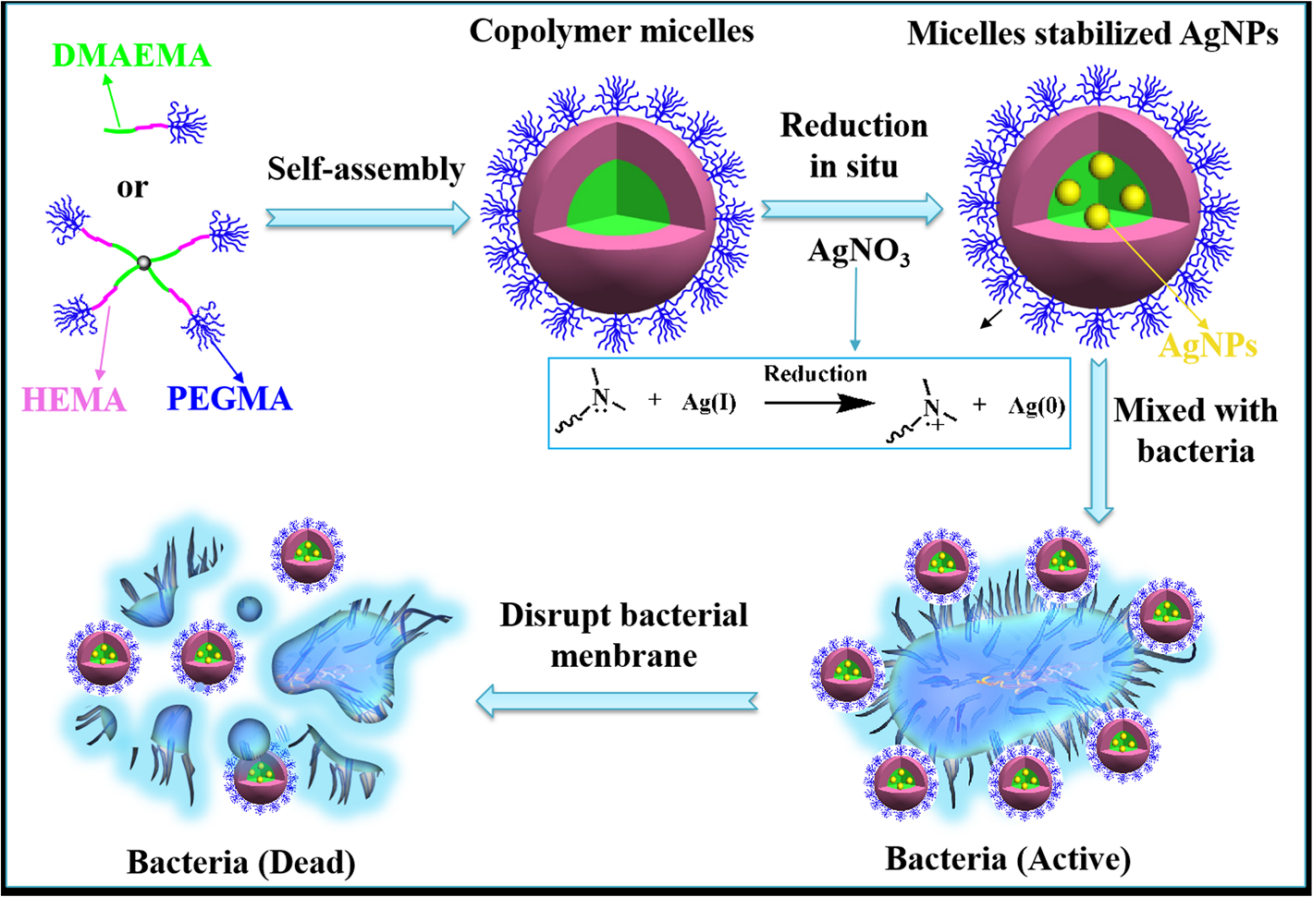
Schematic illustration of the formation of linear/star copolymers micelles stabilized AgNPs for excellent antibacterial activity

In this work, a mild, facile, and green approach has been designed to fight against bacterial infections, taking advantage of polymeric micelles self-assembled from linear or four-arm star triblock copolymers with the similar molecular weight and degree of polymerization as the nanoplatform to decorate AgNPs (Scheme 1). In this approach, the triblock copolymers, composed of DMAEMA, 2-hydroxyethyl methacrylate (HEMA), and PEGMA, could generate self-assembled micelles in aqueous condition, which is a good template for the preparation and the stabilization of AgNPs. The PDMAEMA blocks with tertiary amine groups could easily absorb the Ag^+^ ions by means of coordination interaction and then in situ generate AgNPs without any reducing agent. HEMA and PEGMA blocks with high hydrophilicity could be used as stabilizers in water phase to further improve the stability of AgNPs. Therefore, silver nitrate could spontaneously coordinate and deoxidize onto the nucleus of self-assembled copolymer micelles to form AgNPs. They were embedded in micellar core and could result in the destruction of bacterial membrane. Herein, how linear or four-arm star copolymer topologies affect the maximum absorption wavelength, morphology, particle size, zeta potential, stability, as well as the antibacterial efficiency of AgNPs were under fully investigated. Therefore, the study on the relationship between structure and properties may figure out an in-depth explanation of silver hybrid nanoparticles for the treatment of bacterial infections. In addition, it would provide design ideas and technical basis for the preparation of AgNPs with more stable structure and controllable particle size.

## Material and Methods

### Materials

Pentaerythritol (*J&K* Scientific Ltd.) was dried via reduced pressure for 24 h prior to use. 2-(Dimethylamino) ethyl methacrylate (DMAEMA, > 98%), 2-hydroxyethyl methacrylate (HEMA, 99%), and poly (ethylene glycol) methyl ether methacrylate (PEGMA, *M*_n_ = 300 Da, 99%), all from Aldrich, were purified via passing through a neutral alumina-containing column to remove inhibitor. By the utilization of calcium hydride (CaH_2_), tetrahydrofuran (THF) and toluene from Aldrich were dried and then distilled under reduced pressure before use. Ethyl 2-bromoisobutyrate (EBiB, 98%, Alfa Aesar), 2-bromoisobutyryl bromide (BIBB, 98%, Alfa Aesar), 1,1,4,7,10,10-hexamethyltriethylenetetramine (HMTETA, 99%), silver nitrate (AgNO_3_, 99.9%), cupric bromide (CuBr_2_), methanol, triethylamine (TEA), dichloromethane (DCM), acetone, *n*-hexane, dimethyl sulfoxide (DMSO), stannous octoate (Sn(Oct)_2_), sodium carbonate (Na_2_CO_3_), sodium bicarbonate (NaHCO_3_), sodium chloride (NaCl), sodium sulphate (Na_2_SO_4_), and all other reagents obtained from *J&K* Chemical Company were used as received.

### General Characterization and Instrumentation

Proton nuclear magnetic resonance (^1^H NMR) spectra of the linear or four-arm triblock copolymers were detected in CDCl_3_, and D_2_O at 25 °C through a Bruker ADVANCE 400 MHz spectrometer (Madison, WI, USA). Fourier-transform infrared spectroscopy (FTIR) spectra measurements of linear copolymers, star copolymers, and their micelles stabilized AgNPs were conducted using a FT IR spectrophotometer (Nicolet Nexus for Euro, USA) equipped with a transmission mode at 25 °C. Granular samples were prepared after grinding with potassium bromide (KBr) and then compressing. In order to obtain a spectrum, the spectral conditions were set previously with wavelength from 4000 to 400 cm^−1^ (32 scans) and a resolution of 8 cm^−1^. The zeta potentials of linear and star copolymers micelles stabilized AgNPs at different molar ratios were measured using electrophoretic measurement with the Malvern Zetasizer Nano S instrument (Malvern, WR, UK) in which each sample was tested three times at 25 °C. Transmission electron microscopy (TEM, FEI Tecnai-G20) operating at 200 kV was conducted to observe morphologies of linear and star copolymers micelles stabilized AgNPs at different molar ratios. The preparation process of the product for TEM was as followed: 10 μL sample solution was first dropped on to a copper grid coated with carbon and then dried in the air. UV-Vis spectra of linear and star copolymers micelles stabilized AgNPs at different molar ratios were determined using a UV-Vis spectrophotometer (UV-2450, Shimadzu, Kyoto, Japan). Thermogravimetric analysis (TGA) was performed on a NETZSCH (STA409PC, Germany) equipment. All dried powder samples (linear copolymers, star copolymers, and their micelles stabilized AgNPs) were heated from 25 to 600 °C at a rate of 10 °C /min under nitrogen condition.

### Synthesis of PDMAEMA-*b*-PHEMA-*b*-PPEGMA

The continuous activators regenerated by electron transfer atom transfer radical polymerization (AGERT ATRP) of DMAEMA, HEMA, and PEGMA was performed following the procedure modified from Zhang et al. [29, 30]. In brief, after the addition of CuBr_2_ (10 mg, 0.045 mmol), the 100 mL dry three-necked flask was evacuated and rinsed with argon three times. With the auxiliary of degassed syringe, anhydrous toluene (25 mL), EBiB (88 μL, 0.24 mmol), DMAEMA (5.15 mL, 30.5 mmol), and ligand HMTETA (62 μL, 0.24 mmol) were injected orderly into the container, following 10-min agitation. After injecting Sn(Oct)_2_ (78 μL, 0.24 mmol) with toluene (2 mL) as solution, the reaction was taken place at 70 °C in an oil bath for 8 h. The successive block HEMA (2.32 mL, 18.4 mmol) was injected for next 8-h reaction after the solution became much thicker. At last, with the participation of the third monomer PEGMA (8.89 g, 55.6 mmol), we witnessed a 72-h continuous reaction before cooling the flask to almost room temperature. THF (30 mL) was injected into the container and the reaction mixture was then passed through a neutral alumina column to remove the catalyst. The product PDMAEMA-*b*-PHEMA-*b*-PPEGMA was precipitated into tenfold excess of cold *n*-hexane, filtered, and finally dried under vacuum for 48 h at 35 °C.

### Synthesis of (PDMAEMA-*b*-PHEMA-*b*-PPEGMA)_4_

Star-type bromo-terminated initiator (Br)_4_ was synthesized by the esterification of the terminal hydroxyl groups present on the pentaerythritol with 2-bromoisobutyryl bromide using THF as solvent and TEA as acid-binding agent. Typically, after charging with pentaerythritol (2.72 g, 2 mmol), the 100 mL three-necked flask was evacuated and rinsed with argon three times, following sequential dropwise injection of anhydrous THF (120 mL) and TEA (12.51 mL, 90 mmol). Under the ice/water circumstance, 2-bromoisobutyryl bromide (11.12 mL, 90 mmol) was injected dropwise to the vigorously stirred solution, following 4-h reaction at 0 °C and then 20 h at 25 °C. In order to purify the product, the mixture was first passed through a neutral alumina column. The crude product was washed successively with water, 10% Na_2_CO_3_, saturated NaHCO_3_, and saturated NaCl, then successively dried through Na_2_SO_4_ overnight, filtered, and concentrated before pouring into tenfold excess of cold *n*-hexane to precipitate the product, and finally dried under vacuum for 24 h to receive the product.

The synthetic routes and feeding amounts of (PDMAEMA-*b*-PHEMA-*b*-PPEGMA)_4_ was carried out using the same procedure as PDMAEMA-*b*-PHEMA-*b*-PPEGMA.

### Preparation of AgNPs Using Linear or Star-Shaped Copolymer Micelles

The PDMAEMA-*b*-PHEMA-*b*-PPEGMA or (PDMAEMA-*b*-PHEMA-*b*-PPEGMA)_4_ aqueous solution (pH 7.0) were obtained first, to which the AgNO_3_ solution was added while triggering the reduction reaction of DMAEMA with Ag^+^ to form AgNPs in situ in the micellar core. Taking the mole ratio of DMAEMA and AgNO_3_ equals 9 as an example, firstly, PDMAEMA-*b*-PHEMA-*b*-PPEGMA or (PDMAEMA-*b*-PHEMA-*b*-PPEGMA)_4_ with the same amount of [DMAEMA] = 4.8 mM stirred in acetone (5 mL) for 4 h following by the addition of distilled water (20 mL) under stirring overnight to form stable micelles. Then AgNO_3_ solution (220 μL, 48 mM) was injected dropwise into above solution and stirred at 25 °C in darkness for 48 h. Finally, the linear or star polymeric micelles stabilized AgNPs were prepared by collecting and freeze-drying before storing at − 20 °C for following experiments.

### Antibacterial Assay

The antibacterial investigations on the polymer micelles stabilized AgNPs were carried out against *Escherichia coli* DH5alpha (*E*. *coli* DH5α) strains using Luria-Bertani (LB) medium as carrier to prepare different concentrations of the polymer micelles stabilized AgNPs solutions by ultrasonic. The monoclonal *E*. *coli* DH5α were cultivated overnight in LB medium (5 mL) at 37 °C on a Shaker at 200 rpm before the bacterial suspension was diluted to 1 × 10^5^ CFU/mL by LB medium. After mixing an equal volume of diluted bacterial with different concentrations of copolymer micelles or micelles stabilized AgNPs and incubation at 37 °C for 16 h, the change in the optical density at the wavelength of 600 nm was characterized by a microplate reader (Multiskan Spectrum, Thermo Scientific, Vantaa, Finland). Each assay was repeated six times.

### Cell Viability Evaluation

In order to evaluate the cell viability, the 3-(4,5-dimethylthiazol-2-yl)-2,5-diphenyltetrazolium bromide (MTT) assay with liver hepatocellular carcinoma (HepG2) cells was performed. Prior to cell seeding, HepG2 cells were first incubated in a humidified atmosphere of 5% CO_2_ at 37 °C in Dulbecco’s modified Eagle medium (DMEM) supplemented with 10% fetal bovine serum (FBS), penicillin (100 μL/mL), and streptomycin (0.1 mg/mL). Then, HepG2 cells were seeded in a fresh DMEM medium on 96-well plate at a density of 1 × 10^4^ per well and cultured for 1 day. After substituting DMEM medium in pre-prepared copolymer micelles or micelles stabilized AgNPs solutions (100 μL) at various concentrations, the cells were continued to be cultured for next 24 h. After washed with PBS buffer thrice, the 20 μL MTT reagent (5 mg/mL) and 180 μL fresh DMEM were then added and incubated for another 4 h. Finally, the solution was changed into 200 μL DMSO and the plate was shaken gently for 10 min. The absorbance at 570 nm was measured with above-mentioned microplate reader. The data of six parallel experiments was averaged.

### Dissipative Particle Dynamics Simulation

In order to analyze the growth process of AgNPs, dissipative particle dynamics (DPD) simulation, which was based on the coarse-grained models, was carried out by using the mesocite module of Materials Studio 8.0 (Accelrys Inc., San Diego, CA, USA). As shown in Additional file 1: Figure S1, six kinds of beads constituted the copolymers PDMAEMA-*b*-PHEMA-*b*-PPEGMA or (PDMAEMA-*b*-PHEMA-*b*-PPEGMA)_4_: orange stand for the center, light green for MAA1(methacrylate next to ethylamine side chain), green for DMA (amino ethyl side chain), pink for HEMA, light blue for MAA2 (methacrylate next to PEG side chain), and blue for PEG. A small cluster with unit cell crystal (lattice length: 3.87 Å) consisted of four silver atoms, marked as a silver bead (gold color). At the same time, each water bead (W) in black color contained five water molecules. According to our previous work, Additional file 1: Table S1 showed the result of the computational interaction parameters [31, 32]. A 30 × 30 × 30 *r*_c_^3^ cubic simulation box with periodic boundary conditions was utilized in all directions with 100,000 total simulation steps and 0.05 ns integration time step.

### Statistical Analysis

Statistical analysis was carried out using a two-sample Student’s *t* test with unequal variance. *p* < 0.05 was considered statistically significant.

## Results and Discussion

### Synthesis and Characterization of Linear/Star Copolymers

**Scheme 2:**
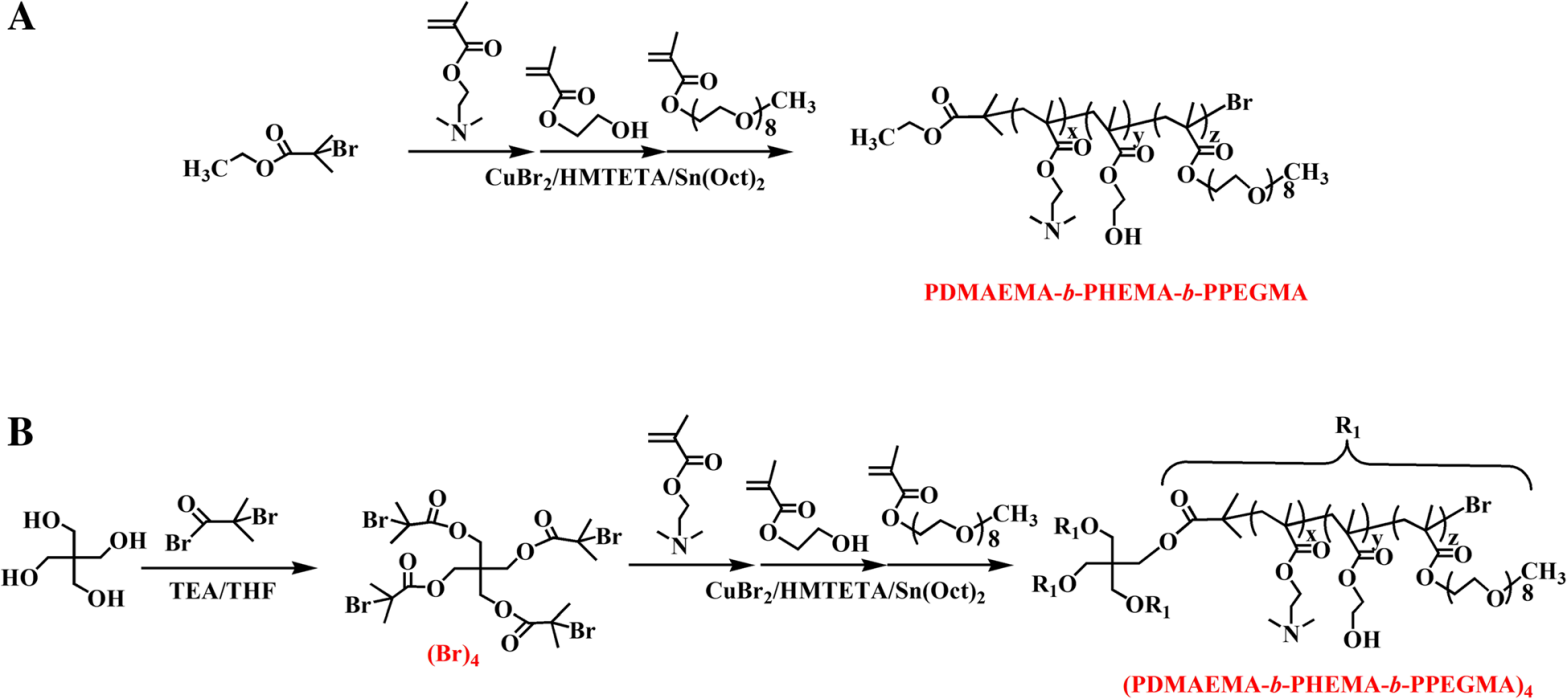
Synthetic routes of **a** PDMAEMA-*b*-PHEMA-*b*-PPEGMA and **b** (PDMAEMA-*b*-PHEMA-*b*-PPEGMA)_4_

Linear copolymers PDMAEMA-*b*-PHEMA-*b*-PPEGMA and star copolymers (PDMAEMA-*b*-PHEMA-*b*-PPEGMA)_4_ were synthesized by ARGET ATRP polymerization of DMAEMA, HEMA, and PEGMA with CuBr_2_/HMTETA as catalyst, Sn(Oct)_2_ as reducing agent, and EBiB or pentaerythritol which was previously acylated with BIBB as initiator in toluene (Scheme 2). The structure and composition of target polymers were verified by ^1^H NMR. Polymeric micelles were self-assembled from PDMAEMA-*b*-PHEMA-*b*-PPEGMA or (PDMAEMA-*b*-PHEMA-*b*-PPEGMA)_4_ with the DMAEMA as the functional block, where the Ag^+^ ions were attracted and reduced into AgNPs without the participation of extra reducing agent. The effects of topological structures of copolymers on AgNPs were discussed and evaluated from the physicochemical properties, antibacterial, and anticancer activities.

The chemical structures of linear/star copolymers were confirmed by ^1^H NMR. First, the end hydroxyl groups of pentaerythritol were entirely transfer to terminal bromine group (Br)_4_, as shown in ^1^H NMR spectrum (Additional file 1: Figure S2). The peak at 4.33 ppm was attributed to the -C*H*_2_O- in pentaerythritol, while the new -(C*H*_3_)_2_- signal at 1.94 ppm appeared. And the integration ratio values of the peaks at 1.94 ppm to 4.33 ppm were around 3. As shown in Additional file 1: Figure S3 and S4, the peak of -C(C*H*_3_)_2_- appeared at 1.94 ppm. The signals at 1.83 ppm, 1.00 ppm were assigned to -C*H*_2_- and -CC*H*_3_- in the main chains of methyl methacrylate, respectively. The peaks at 2.58 ppm and 4.08 ppm belonged to the characteristic resonances of the coterminous two methylene protons -C*H*_2_C*H*_2_- in DMAEMA block, and the peak at 2.29 ppm was assigned to methyl protons -C*H*_3_-, which was attached to a tertiary amine group. The presence of -C*H*_*2*_C*H*_*2*_- methylene protons connected to the terminal hydroxyl group in the HEMA unit appeared at 4.08 ppm and 3.57 ppm, respectively. The characteristic PEG peaks of -OC*H*_2_C*H*_2_- and terminal -C*H*_3_ protons appeared at 3.67 and 3.39 ppm, respectively. Double bond peaks at 5.5–6.1 ppm disappeared in the ^1^H NMR spectra. Calculated from the integration ratio values of signal (f) to (a) (*I*_f_/*I*_a_), signal (g) to (a) (*I*_g_/*I*_a_), and signal (h) to (a) (*I*_h_/*I*_a_), respectively, PDMAEMA_19.3_-*b*-PHEMA_12.5_-*b*-PPEGMA_24.6_ and (PDMAEMA_5.0_-*b*-PHEMA_5.6_-*b*-PPEGMA_5.0_)_4_ were obtained.

### Preparation and Characterization of the Linear/Star Copolymer Micelles Stabilized AgNPs

The formation process of AgNPs was shown in Scheme 1. A lone pair of electrons on the N atom which belong to the tertiary amine group in PDMAEMA molecular chain, owning a capability of coordination and reduction, thus it could be used both as a trapping agent and a reducing agent. First, silver ions were trapped by PDMAEMA because of the complexation between Ag^+^ and N atom, forming (Ag^+^)-PDMAEMA complex. Subsequently, Ag^+^ were reduced in situ to form silver atoms at the nucleation stage. Afterwards, the nucleation of Ag continued with the growth of Ag crystal, resulting in the formation of AgNPs [26]. The hydrophilic block PPEGMA acted as micellar shell, which provided a steady protective layer and further improved the stability of AgNPs. The steric stabilization effect of self-assembled micelles within the system was determined by the thermodynamic equilibrium of micelles on the stabilization of AgNPs and the agglomeration between AgNPs. In case of a small amount of AgNPs, the steric stabilization of the copolymer could prevent further aggregation of the AgNPs. With the number of Ag increasing, the stability of the micelles to the AgNPs would be weakened and then the possibility of collision between particles would rise, resulting in the size growth of AgNPs. Taking advantage of the spatial stabilization of micelles, the AgNPs we prepared have controlled particle size, which is of great potential for antibacterial applications.

**Fig. 1:**
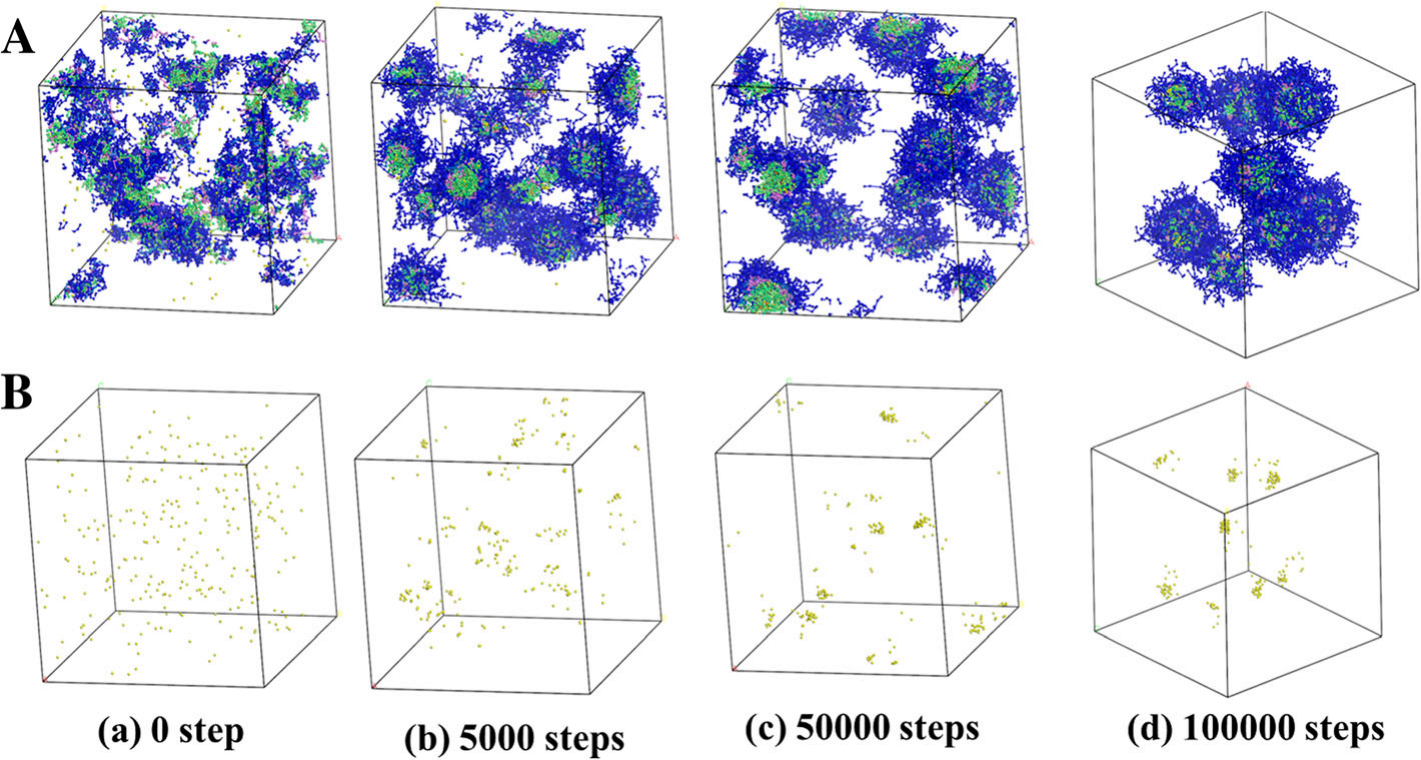
DPD simulation of the growth process and distribution of AgNPs with PDMAEMA-*b*-PHEMA-*b*-PPEGMA at PDMAEMA/AgNO_3_ molar ratio = 1/1 in different simulation time. **a** The water molecules were hidden for clarity. **b** Only the AgNPs were shown

DPD simulation was performed to investigate the growth process and distribution of AgNPs, with the same concentrations of actual experiment (PDMAEMA/AgNO_3_ molar ratio = 1/1, the volume fractions of linear copolymers, Ag and water beads being 10%, 0.23%, and 89.77%, respectively). Figure 1 revealed that the beads of PDMAEMA-*b*-PHEMA-*b*-PPEGMA and AgNPs were initially showed an irregularly distributed state in aqueous solution. As time went on, eight self-assembled copolymer micelles were finally formed and dispersed uniformly, while all the Ag beads were loaded into the micelles. It could be seen that AgNPs in equilibrium could be stabilized in the copolymer micelles without further aggregation, indicating that the self-assembled micelles were able to prevent the further aggregation of AgNPs and then achieved the purpose of controlling their particle size and distribution.

**Fig. 2:**
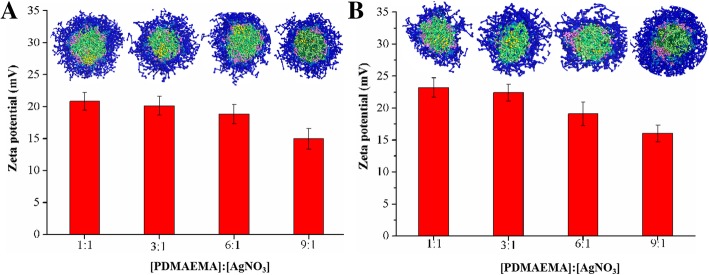
The zeta potentials and the cross-section views of **a** linear and **b** star copolymers micelles stabilized AgNPs. The data was collected with different PDMAEMA/AgNO_3_ molar ratios of (a) 1/1, (b) 3/1, (c) 6/1, (d) 9/1

The FT IR characterization of linear/star copolymers and their micelles stabilized AgNPs were shown in Additional file 1: Figure S5. Obviously, compared with the simple linear/star copolymers, the -COOR stretching vibration at 1730 cm^−1^ and the bending vibration of C-N bond in PDMAEMA at 1457 cm^−1^ decreased after AgNPs formation, indicating that the AgNPs have been successfully loaded onto copolymer micelles. The crystalline nature of linear/star copolymers micelles stabilized AgNPs was confirmed by X-ray diffraction spectrum (Additional file 1: Figure S6). The diffraction peaks values of 38.5°, 44.8°, 64.2°, and 78.0° were corresponded to the (111), (200), (220), and (311) crystal faces of the face-centered cubic (fcc)crystal structure of Ag-contained nanoparticles [33, 34]. The zeta potentials of linear/star copolymers micelles stabilized AgNPs were measured. As shown in Fig. 2, the zeta potentials of these copolymer micelles stabilized AgNPs were around 15.0–23.2 mV. Moreover, with the increasing amount of AgNO_3_, the zeta potential of micelles stabilized AgNPs remarkably increased because of the decoration of more AgNPs. To further investigate the dispersion of AgNPs and the stabilizing effect of micelles on AgNPs, DPD simulations of linear/star copolymers micelles stabilized AgNPs at different PDMAEMA/AgNO_3_ molar ratios were carried out. As shown in Fig. 2, the results also demonstrated that the sizes of AgNPs were proportional to the ratios where the number of aggregated small AgNPs increased and the distance between them decreased, leading to the rising probability of collision and agglomeration.

**Fig. 3:**
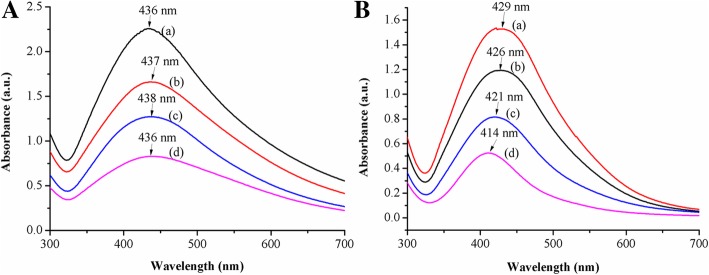
UV-Vis spectra of **a** linear and **b** star copolymers micelles stabilized AgNPs at different PDMAEMA/AgNO_3_ molar ratios of (a) 1/1, (b) 3/1, (c) 6/1, (d) 9/1

The spectra for four linear copolymers micelles stabilized AgNPs owned minor difference at the maximum absorption peak located around 437 nm, which was characteristic surface plasmon resonance (SPR) absorption peak of spherical/near-spherical AgNPs, related to both resonance excitation and interband transition of them (Fig. 3a). The result proved that the tertiary amine group in the linear copolymers could react with silver nitrate and the formation of AgNPs hardly depended on the steric hindrance of the linear copolymers micelles. Subsequently, star copolymers with the similar block and polymerization degree under the same conditions, the particle size of AgNPs decreased when the PDMAEMA/AgNO_3_ molar ratio increased. It was reflected through the hypsochromic shift in the UV-Vis spectra where the maximum absorption peaks were at 429 nm, 426 nm, 421 nm, and 414 nm, respectively, due to the different quantity of AgNPs formed by coordination reduction on the tertiary amine of star copolymer micelles (Fig. 3b). In other words, the steric stabilization of the star copolymers could stabilize the AgNPs better and prevent its further aggregation at small amount of AgNPs. In contrast, the increase in the amount of AgNPs weakened the stabilizing effect, which provided more opportunities to the collision of AgNPs, and finally resulted in larger AgNPs. Comparing Fig. 3a with Fig. 3b, the absorption peaks at 437 nm of AgNPs within linear copolymer micelles owned wider wavelength distribution, while AgNPs within star copolymer micelles were at around 422 nm. Herein, there was no blue shift shown in the spectra of the linear copolymers, which could be explained by the fact that the blocks of linear copolymers micelles have weaker effect on the steric hindrance for AgNPs, which resulted in increasing the probability of collisional agglomeration between AgNPs.

**Fig. 4:**
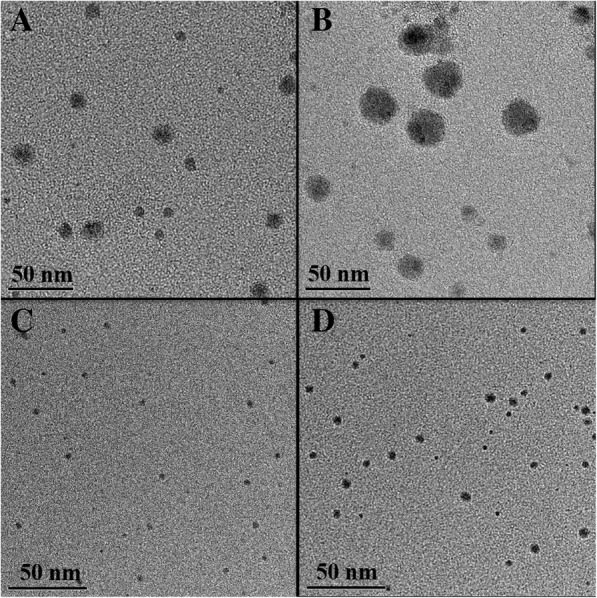
TEM image of **a**, **b** linear copolymers and **c**, **d** star copolymers micelles stabilized AgNPs at different PDMAEMA/AgNO_3_ molar ratios: **a**, **c** 6/1, **b**, **d** 1/1

TEM measurements were then carried out to determine the size, size distribution, and morphology of the AgNPs. The TEM images of AgNPs depended on AgNO_3_ feeding ratios were shown in Fig. 4. When the PDMAEMA/AgNO_3_ molar ratios were 6 and 1, calculated using ImageJ software, the particle size of linear copolymers micelles stabilized AgNPs were 11.1 nm and 25.7 nm, while the diameter of star copolymers micelles stabilized AgNPs were 3.7 nm and 6.4 nm, respectively. The increasing of AgNO_3_ content led to more silver atoms in micelles, higher surface energy, and the number of aggregated AgNPs increases accordingly with a larger AgNPs size. It was clear that micelles stabilized AgNPs were monodisperse and spherical with somewhat uneven of linear copolymer micelles stabilized AgNPs. The sizes of micelles stabilized AgNPs were further complement the UV-Vis results.

### Stability of the Linear/Star Copolymers Micelles Stabilized AgNPs

**Fig. 5:**
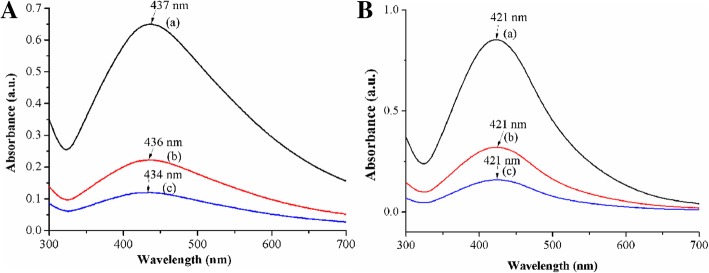
UV-Vis spectra of **a** linear copolymers and **b** star copolymers micelles stabilized AgNPs solution at PDMAEMA/AgNO_3_ molar ratio = 6/1 after 1 month at the diluted times of 1 (a), 3 (b), and 6 (c), respectively

The stability of the linear/star copolymers micelles stabilized AgNPs is of great influence for the development of biomedical field. Obviously, the SPR peak in UV-Vis spectra (Fig. 5) of star copolymer micelles stabilized AgNPs did not display any significant changes for at least 1 month even after further diluted by one time, three times, and six times, indicating that the prepared AgNPs appeared well long-term colloidal stability within the experimental concentration range. However, the results of linear copolymer micelles stabilized AgNPs showed that the UV absorption wavelength decreased slightly as the increase of dilution ratios. And the micelles concentration of linear copolymer decreased after 1 month of placement may lead to insufficient provision of steric hindrances to stabilize AgNPs.

**Fig. 6:**
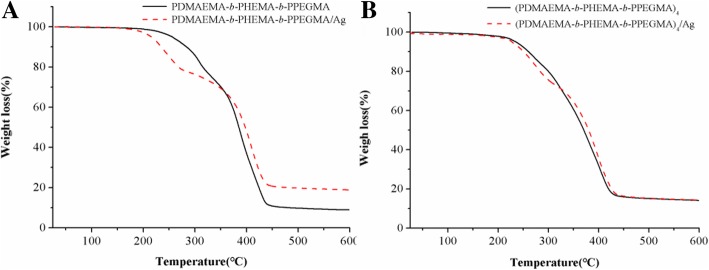
TGA curves of **a** linear copolymers and **b** star copolymers micelles and their micelles stabilized AgNPs at PDMAEMA/AgNO_3_ molar ratio = 6/1

From the thermogravimetric analysis curves in Fig. 6, it was shown that the initial decomposition temperature (*T*_onset_) of linear copolymers micelles was 217 °C, which shifted to 172 °C after silver loading, suggesting that the linear copolymer micelles stabilized AgNPs showed lower thermal stability than the pure linear copolymers micelles. It may be due to the fact that the chemical structure of PDMAEMA in the molecular chain changes and the catalytic effect of AgNPs in the thermal degradation process cannot be ignored [35]. As for star copolymers and their stabilized AgNPs, *T*_onset_ were around 213 °C. The two T_onset_ of star copolymers micelles and their micelles stabilized AgNPs showed very few gaps, which could be speculated that the more stable star-shaped copolymers have better effect on stabilizing AgNPs than the linear copolymers. Combined the results of UV-Vis, TEM, and TGA measurements, it could be inferred that compared to the linear copolymers, the star copolymers have superior advantages in topology for stabilizing AgNPs, such as better stability, more uniform dispersion, slower nucleation rate during reduction, and the better product with a smaller and more uniform size of AgNPs.

### Antibacterial Activity and Cell Viability

**Fig. 7:**
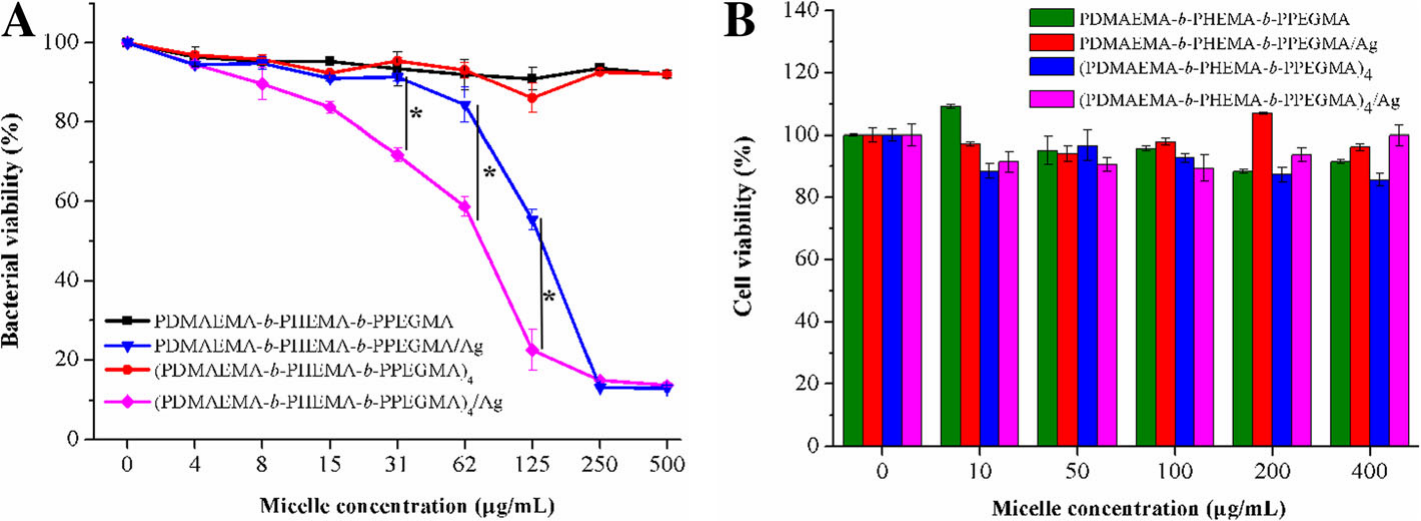
**a** Antibacterial activity and **b** cell viability of linear copolymers and star copolymers micelles stabilized AgNPs at PDMAEMA/AgNO_3_ molar ratio = 6/1. *p < 0.05, two-tailed Student *t* test

To evaluate the antibacterial activities of the linear/star copolymers micelles stabilized AgNPs by optical density (OD_600_) measurements, *E*. *coli* DH5α was selected as the Gram-negative bacterial model. The absorbance at 600 nm after incubation was tested by incubating the bacteria with the eight different concentrations of micelles and micelles stabilized AgNPs at 37 °C. Results shown in Fig. 7a illustrated that the bacterial growth curves were highly correlated with the AgNPs concentration in the LB medium. The inhibition of linear/star copolymers micelles on the growth of bacteria was weak, which was not fatal to bacteria. However, as the concentration of linear/star copolymers micelles stabilized AgNPs increased, the survival rate of *E*. *coli* DH5α was significantly inhibited, indicating a strong antibacterial efficacy of AgNPs against *E*. *coli* DH5α. The concentrations of linear copolymers micelles stabilized AgNPs preventing the bacterial growth in the experiments were relatively higher than those of star copolymers micelles stabilized AgNPs, which might due to the fact that bigger size of AgNPs could lead to a lower antibacterial performance because of the inefficient exposure of bacteria to AgNPs and relatively slow release behavior of AgNPs.

Cancer is an uncontrollable disease of cell growth that can occur in any part of the body. The most common cancers are liver cancer, breast cancer, colorectal cancer, and lung cancer. Among them, the liver cancer has the much higher prevalence in both developed and developing countries. Therefore, the toxicity experiments of the linear/star copolymers micelles stabilized AgNPs on HepG2 cells were carried out, in which HepG2 cells were incubated with linear/star copolymers micelles stabilized AgNPs at different concentrations (10, 50, 100, 200, 400 mg/L, respectively) for 48 h and the cell viability with MTT assay was the most intuitive data to evaluate the biocompatibility of the composite material. As shown in Fig. 7b, the percentage of viable cells for the linear/star copolymers micelles stabilized AgNPs exhibited negligible cytotoxicity, and was about 90% viability even at the highest concentration applied (400 μg/mL) after 48-h incubation, indicating the advantageous cytocompatibility of the micelles stabilized AgNPs within a relatively wide range of concentration.

## Conclusion

In conclusion, PDMAEMA-based linear and star copolymer micelles as effective delivery carriers for silver-bearing antimicrobials were developed, and their in vitro antimicrobial efficacy and cell viability were investigated. Being a reducing agent and a stabilizer simultaneously, the micellar PDMAEMA core acted as loading platform for AgNPs in situ translated from the precursor silver nitrate. In silico simulation and experimental results indicated that both types of the copolymer micelles could generate monodisperse and spherical AgNPs. Compared with linear copolymers sliver-bearing micelles, the fabricated star copolymers micelles stabilized AgNPs exhibited smaller average size, better stability against dilution and pyrogenic decomposition, and enhanced antibacterial activities against *E*. *coli* DH5α due to the serious damage of bacterial membrane caused by loaded AgNPs. Moreover, both types of copolymer micelles stabilized AgNPs possessed great cytocompatibility toward HepG2 cells. Therefore, these studies may provide some guidance for the construction of more effective AgNPs weapon with well-defined and feasible polymer topology for combating the multiple bacteria-induced infections.

## Additional file

Additional file 1: **Table S1.** DPD bead interaction parameters *a*_*ij*_. **Figure S1.** The molecular structures and coarse-grained models of (I) PDMAEMA-*b*-PHEMA-*b*-PPEGMA or (PDMAEMA-*b*-PHEMA-*b*-PPEGMA)_4_, (II) Ag, and (III) Water. **Figure S2.** ^1^H NMR spectrum of (Br)_4_. **Figure S3.** ^1^H NMR spectrum of the linear copolymer PDMAEMA-*b*-PHEMA-*b*-PPEGMA. **Figure S4.** ^1^H NMR spectrum of the star copolymer (PDMAEMA-*b*-PHEMA-*b*-PPEGMA)_4_. **Figure S5.** FT IR spectra of (A) linear copolymers and (B) star copolymers: (a) linear/star copolymers and (b) their micelles stabilized AgNPs at PDMAEMA/AgNO_3_ molar ratio of 6/1. **Figure S6.** XRD patterns of (A) linear and (B) star copolymers micelles stabilized AgNPs at PDMAEMA/AgNO_3_ molar ratio of 6/1. (DOC 5709 kb)

## Data Availability

The datasets used and/or analyzed during the current study are available from the corresponding author on reasonable request.

## Abbreviations

AgNPs: Silver nanoparticles
DMAEMA: 2-(dimethylamino) ethyl methacrylate
HEMA: 2-hydroxyethyl methacrylate
PEGMA: Poly (ethylene glycol) methyl ether methacrylate
CuBr_2_: Cupric bromide
^1^H NMR: Proton nuclear magnetic resonance
FTIR: Fourier-transform infrared spectroscopy
KBr: Potassium bromide
UV-Vis: Ultraviolet-visible
MTT: 3-(4,5-Dimethylthiazol-2-yl)-2,5-diphenyltetrazolium bromide
HepG2: Liver hepatocellular carcinoma
DPD: Dissipative particle dynamics
SPR: Surface plasmon resonance
XRD: X-ray diffraction
