# Supplementary material for: Facile In Situ Preparation and In Vitro Antibacterial Activity of PDMAEMA-Based Silver-Bearing Copolymer Micelles
Source: Nanoscale Res Lett. 2019 Jul 27;14:256. doi: 10.1186/s11671-019-3074-z (PMC6661048; doi:10.1186/s11671-019-3074-z)
Supplement: Supplementary file 1 — Table S1. DPD bead interaction parameters aij. Figure S1. The molecular structures and coarse-grained models of (I) PDMAEMA-b-PHEMA-b-PPEGMA or (PDMAEMA-b-PHEMA-b-PPEGMA)4, (II) Ag, and (III) Water. Figure S2. 1H NMR spectrum of (Br)4. Figure S3. 1H NMR spectrum of the linear copolymer PDMAEMA-b-PHEMA-b-PPEGMA. Figure S4. 1H NMR spectrum of the star copolymer (PDMAEMA-b-PHEMA-b-PPEGMA)4. Figure S5. FT IR spectra of (A) linear copolymers and (B) star copolymers: (a) linear/star copolymers and (b) their micelles stabilized AgNPs at PDMAEMA/AgNO3 molar ratio of 6/1. Figure S6. XRD patterns of (A) linear and (B) star copolymers micelles stabilized AgNPs at PDMAEMA/AgNO3 molar ratio of 6/1. (DOC 5709 kb) [file 11671_2019_3074_MOESM1_ESM.doc]

**Additional file for**

**Facile *in situ* preparation and *in vitro* antibacterial activity of PDMAEMA-based silver-bearing copolymer micelles**

Wenjing Lin^1^, Kaihang Huang^1^, Yanzhe Li^1^, Yanlin Qin^1^, Di Xiong^3^, Jiabao Ling^1^, Guobin Yi^1^, Zilun Tang^1^, Jinglian Lin^2^, Yunwei Huang^1^, Chufen Yang^1,*^, Jufang Wang^2,*^

^1^ School of Chemical Engineering and Light Industry, Guangdong University of Technology, Guangzhou 510006, PR China

^2^ School of Bioscience & Bioengineering, South China University of Technology, Guangzhou 510640, P R China

^3^ School of Chemical Engineering, Xiangtan University, Xiangtan 411105, China

**Table S1** DPD bead interaction parameters *a_ij_*.

|  | Ag | DMA | MAA1 | Center | HEMA | MAA2 | PEG | Water |
| --- | --- | --- | --- | --- | --- | --- | --- | --- |
| Ag  DMA  MAA1 | 15.00  28 | 25.00  25.78 |  |  |  |  |  |  |
|  | 33 |  | 25.00 |  |  |  |  |  |
| Center | 28 | 25.52 | 25.35 | 25.00 |  |  |  |  |
| MAA2 | 33 | 27 | 25.68 | 28.94 | 25.00 |  |  |  |
| MAA3 | 33 | 31 | 26.8 | 34.04 | 26.50 | 25.00 |  |  |
| PEG | 50 | 46 | 36.48 | 53.24 | 32.56 | 28.48 | 25.00 |  |
| Water | 110 | 60 | 56 | 81.94 | 52 | 48 | 26.10 | 25.00 |


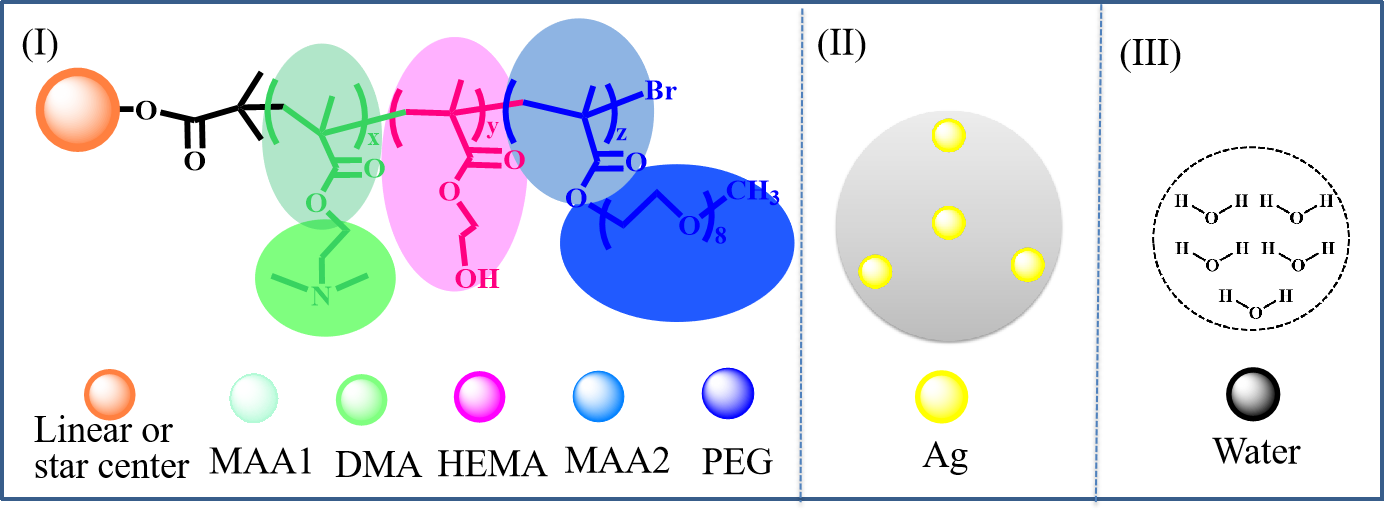


**Figure S1.** The molecular structures and coarse-grained models of (I) PDMAEMA-*b*-PHEMA-*b*-PPEGMA or (PDMAEMA-*b*-PHEMA-*b*-PPEGMA)_4_, (II) Ag, and (III) Water.





**Figure S2.** ^1^H NMR spectrum of (Br)_4_.

**
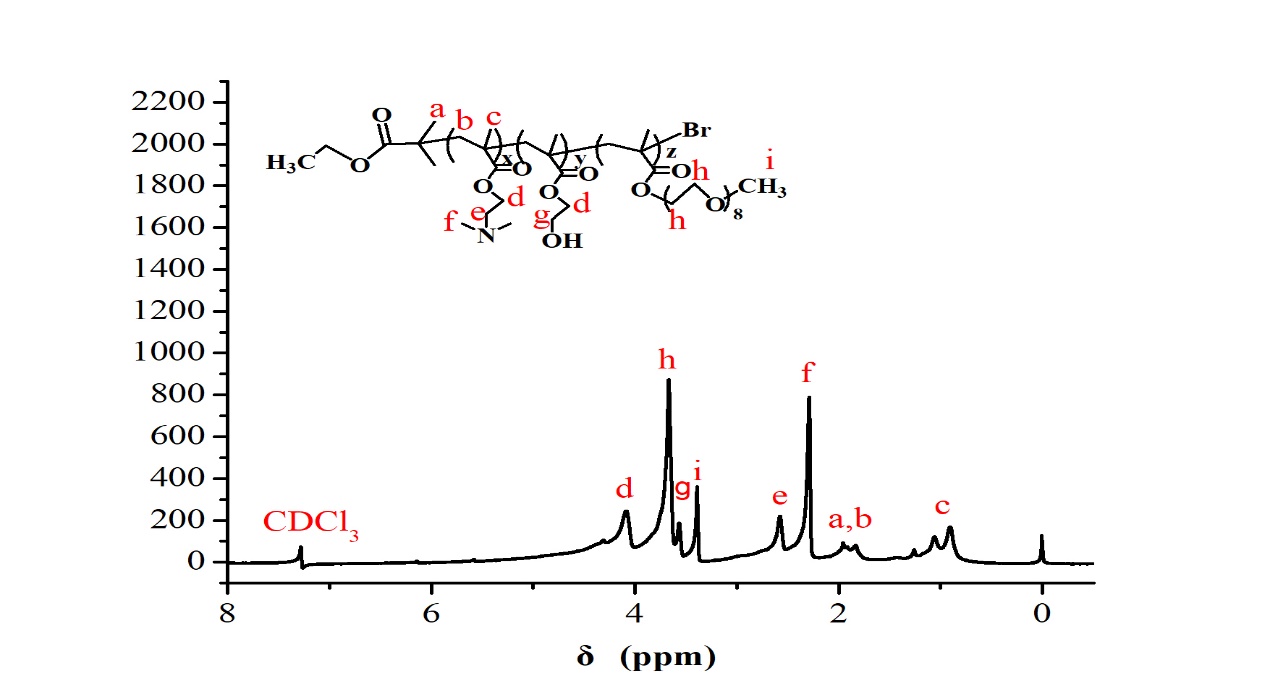
**

**Figure S3.** ^1^H NMR spectrum of the linear copolymer PDMAEMA-*b*-PHEMA-*b*-PPEGMA.





**Figure S4.** ^1^H NMR spectrum of the star copolymer (PDMAEMA-*b*-PHEMA-*b*-PPEGMA)_4_.


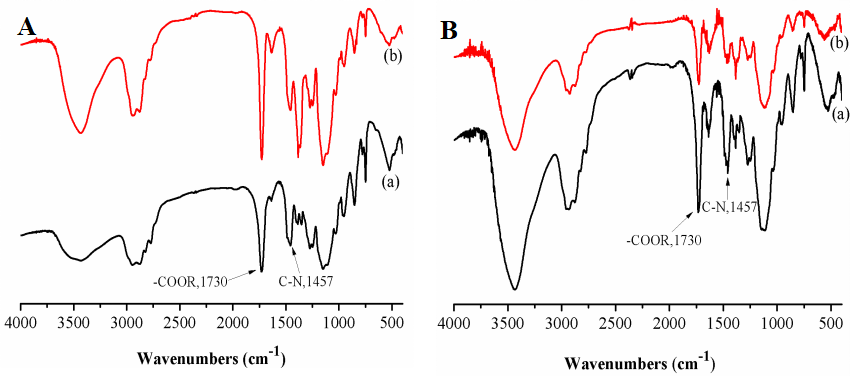


**Figure S5.** FT IR spectra of (A) linear copolymers and (B) star copolymers: (a) linear/star copolymers and (b) their micelles stabilized AgNPs at PDMAEMA/AgNO_3_ molar ratio of 6/1.


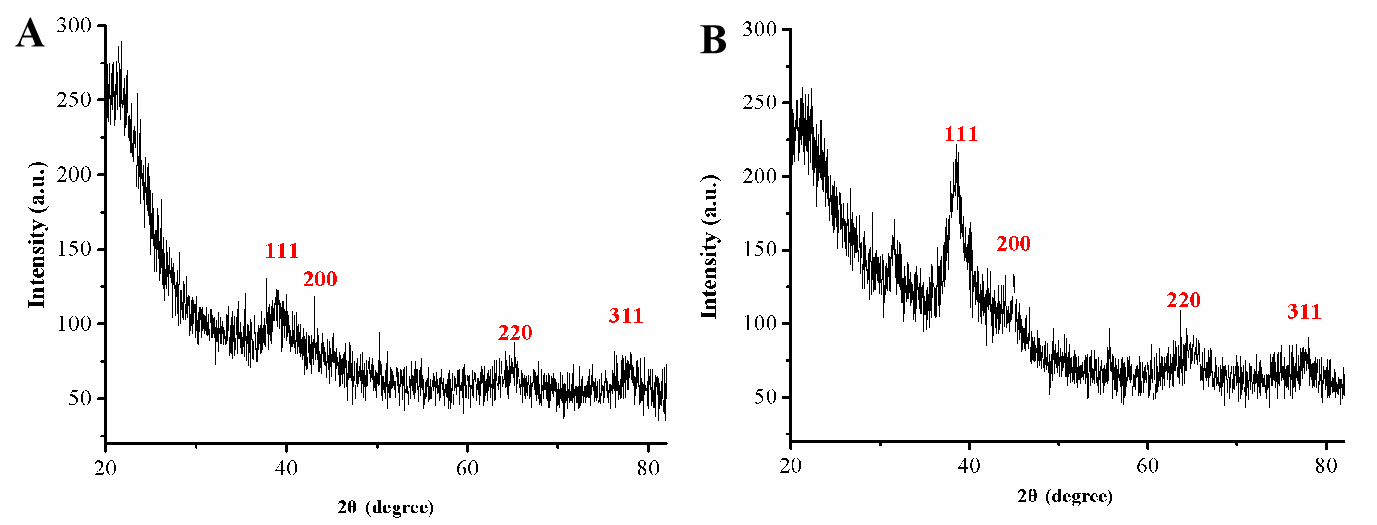


**Figure S6.** XRD patterns of (A) linear and (B) star copolymers micelles stabilized AgNPs at PDMAEMA/AgNO_3_ molar ratio of 6/1.

.
